# Morphology and phylogeny of two acotylean flatworms (Platyhelminthes, Polycladida) from the genera *Cryptophallus* and *Limnoplana* in the South China Sea

**DOI:** 10.3897/zookeys.1275.180665

**Published:** 2026-03-30

**Authors:** Hai-Long Liu, Bing-Bing He, Shuang-Fei Li, An-Tai Wang, Zhang-Li Hu, Yu Zhang

**Affiliations:** 1 Shenzhen Key Laboratory of Marine Bioresource and Eco-environmental Science, Guangdong Engineering Research Center for Marine Algal Biotechnology, College of Life Sciences and Oceanography, Shenzhen University, Shenzhen, China College of Life Sciences and Oceanography, Shenzhen University Shenzhen China; 2 Key Laboratory of Optoelectronic Devices and Systems of Ministry of Education and Guangdong Province, College of Physics and Optoelectronic Engineering, Shenzhen University, Shenzhen, China College of Physics and Optoelectronic Engineering, Shenzhen University Shenzhen China

**Keywords:** Brackish water, marine flatworm, molecular phylogeny, new species, polyclad, systematics, taxonomy

## Abstract

We describe two new species of the genera *Cryptophallus* Bock, 1913 and *Limnoplana* Faubel, 1983, which have not been reported for nearly a century. Herein, the genus *Limnoplana* represents a rare group of polyclads inhabiting brackish waters. *Cryptophallus sinensis***sp. nov**. is characterized by: i) a grayish-brown dorsal surface with a slightly darker median region, ii) numerous cerebral eyespots forming two scarcely separate clusters, and iii) an ejaculatory duct joining the prostatic duct at the distal end of the penis. *Limnoplana obscuriviridis***sp. nov**. is distinguished from its congeners by: i) the presence of a true seminal vesicle, ii) a heart-shaped brain, iii) gonopores positioned closer to the posterior end of the body, and iv) a U-shaped Lang’s vesicle with two swollen ends. We provide the first partial DNA maker sequences for these two new species and employ them to analyze the phylogenetic relationships of their corresponding genera. Our molecular phylogenetic analyses show that *Cryptophallus* and *Limnoplana* are nested within the Stylochoidea clade. In addition, we have revised the genus *Cryptophallus*; its familial affiliation remains unresolved, and additional species are required to clarify its phylogenetic position.

## Introduction

Polycladida are free-living platyhelminthes, characterized by a highly branched intestine, from which their name is derived (Hyman 1951; Quiroga et al. 2004). All polyclads inhabit marine environments, except for three brackish-water species in the family Limnostylochidae Faubel, 1983 (Kaburaki 1918; Faubel 1983). Polyclads generally feed on other invertebrates and play an important predatory role in hard substrate environments (Newman et al. 2000; Rawlinson 2007). Due to the relatively limited number of studies and specialists focused on this group, coupled with challenges in specimen collection, handling, and identification (Rawlinson 2007; Bahia et al. 2012), it is expected that many undescribed species remain to be discovered.

The acotylean polyclad genera *Cryptophallus* Bock, 1913 and *Limnoplana* Faubel, 1983 are rarely recorded, with most species described more than a century ago. Bock (1913) established the genus *Cryptophallus* within Stylochidae Stimpson, 1857, which is characterized by: a broadly oval body with a pair of small knob-like tentacles; numerous cerebral eyes arranged in single cluster or two indistinct clusters; marginal eyes distributed around the entire body margin; a mouth situated at the posterior part of the pharynx; male copulatory apparatus beneath the pharyngeal pouch, with spermiducal bulbs, a small prostatic vesicle and an unarmed penis papilla; and a ductus vaginalis and vagina externa opening into a common pore. Later, Faubel (1983) reassigned species of *Cryptophallus* to the families Callioplanidae Hyman, 1953 and Pseudostylochidae Faubel, 1983 based on the lining of the prostatic vesicle; however, we propose that this classification may be inappropriate (see Remarks section of *Cryptophallus*).

The family Limnostylochidae is characterized by i) a ruffled pharynx positioned in the anterior half of the body; ii) an elongate, tube-like prostatic vesicle with smooth glandular lining; and iii) occurrence in limnic or brackish-water habitats. This family comprises three species, divided into two genera: *Limnoplana* and *Limnostylochus* Bock, 1913. *Limnoplana* is distinguished from *Limnostylochus* by the presence of an unarmed penis papilla and the absence of a seminal vesicle and spermiducal bulbs (Faubel 1983).

Traditional polyclad taxonomy relies primarily on morphological traits, including body coloration pattern, eye arrangement, tentacle morphology, pharynx structure, and reproductive anatomy (Hyman 1953; Faubel 1983, 1984; Prudhoe 1985). In recent years, molecular phylogenetic studies have provided new insights, clarifying the evolutionary relationships among several major lineages and resolving some of the incongruences observed in morphology-based phylogenies (Aguado et al. 2017; Bahia et al. 2017; Tsunashima et al. 2017; Dittmann et al. 2019; Litvaitis et al. 2019; Oya and Kajihara 2020; Rodríguez et al. 2021). However, molecular data remain unavailable for most species (in some cases, for entire families or genera), which limits a comprehensive understanding of polyclad phylogenetic relationships.

In this study, we describe two new species of the genera *Cryptophallus* and *Limnoplana* from China. We also provide the first molecular data for these two genera and infer their phylogenetic positions among other polyclads, via molecular analyses.

## Material and methods

Specimens were collected from two sites in Guangdong Province, China. Live individuals were anesthetized using an MgCl_2_ solution prepared with tap water and adjusted to match the salinity of the respective habitat (seawater or brackish water), and then photographed using a Leica MZ16 stereomicroscope (Leica Microsystems, Germany). A part of each specimen was fixed with 95% ethanol for molecular analysis, while the remaining part was fixed with 10% formalin in seawater for histological study. For histological examination, specimens were dehydrated in an ethanol series, cleared in xylene, and subsequently embedded in paraffin wax. Serial sections were cut at intervals of 7 µm and were stained with modified Cason’s Mallory-Heidenhain stain solution (Yang et al. 2020), which provides better visualization of secretions and connective tissues compared to the conventional hematoxylin and eosin (H&E) staining method. Images were captured using a Nikon DS-Ri2 digital camera under a Nikon ECLIPSE Ni compound microscope (Nikon, Japan). All histological preparations have been deposited in the Marine Biological Museum, Chinese Academy of Sciences (MBMCAS), Qingdao, China.

Total DNA was extracted using the DNeasy Blood & Tissue Kit (QIAGEN, Germany). Fragments of the 18S rDNA (18S), 28S rDNA (28S), 16S rDNA (16S), and cytochrome *c* oxidase subunit I (COI) genes were amplified by polymerase chain reaction (PCR). Primers used in this study are listed in the Suppl. material 1: table SS1. Thermal cycling was initiated with 3 min at 94 °C, followed by 35 cycles of denaturation at 94 °C for 45 s, annealing at gene-specific temperature (49 °C for COI, 48 °C for 16S, and 52 °C for 28S and 18S) for 45 s, and extension at 72 °C for 1 min. The cycling ended with a final extension at 72 °C for 7 min. The amplified products were sequenced by RuiBiotech (Guangzhou, China) using double-stranded Sanger sequencing to verify accuracy. Sequences were checked and edited using SeqMan software (DNAStar Inc., Madison, USA), and the final edited sequences have been deposited in GenBank.

A total of 70 terminal taxa were included in the phylogenetic analyses (Suppl. material 1: table S2). *Cestoplana rubrocincta* (Grube, 1840) and *Pericelis tectivorum* Dittmann et al., 2019 were used as outgroups. Sequences were aligned using MAFFT ver. 7 (Katoh and Standley 2013) with the “Auto” option. Ambiguous sites were removed using Gblocks (Talavera and Castresana 2007) with the following parameter settings: minimum number of sequences for a conserved/flank position (24/24), maximum number of contiguous non-conserved positions (8), minimum length of a block (10), and allowed gap positions (half). TIM3e+R2 (18S), TIM3+F+I+G4 (28S), TVM+F+I+G4 (16S), GTR+F+I+G4 (COI) were selected as the best-fit model by using ModelFinder (Kalyaanamoorthy et al. 2017) according to the Bayesian Information Criterion (BIC). We used IQ-TREE 2 (Minh et al. 2020) to infer the maximum-likelihood tree using the edge-linked partition model. Maximum-likelihood trees were inferred with 5000 ultrafast bootstrap replicates. Bayesian inference (BI) was performed using MrBayes ver. 3.2.2 (Ronquist et al. 2012). MrBayes was run for 20 million generations with tree sampling every 1000 generations, two parallel runs and four independent Markov chains per run. All model parameters (nst = 6; rates = invgamma) were unlinked among partitions. The standard deviation of the split frequencies (< 0.01) is used as the criterion to validate the convergence of the analysis.

## Results

### Taxonomy

#### Cryptophallus

Taxon classificationAnimaliaPolycladidaPseudostylochidae

Bock, 1913

78AC0032-E967-5B52-ADD3-9C232EBD40D1

##### Type species.

*Cryptophallus wahlbergi* Bock, 1913.

##### Emended diagnosis.

*Cryptophallus* is characterized by: i) the presence of small knob-like tentacles; ii) the presence of tentacular, cerebro-frontal, and marginal eyespots; iii) the male copulatory apparatus not enclosed by a muscular bulb and situated beneath the posterior end of the pharynx, with spermiducal bulbs and a small prostatic vesicle, the latter lined with a smooth or slightly folded epithelium and positioned vertically above a wide, conical, unarmed penis papilla; and iv) ductus vaginalis and vagina externa opening into a common pore.

##### Remarks.

In its original description, the genus *Cryptophallus* included seven species: *Cryptophallus wahlbergi* Bock, 1913, *Cryptophallus bartschi* Kaburaki, 1923, *Cryptophallus sondaicus* Bock, 1925, *Cryptophallus magnus* Freeman, 1933, *Cryptophallus eximius* Kato, 1937, *Cryptophallus aegypticus* Melouk, 1940, and *Cryptophallus japonicus* Kato, 1944. *Cryptophallus magnus* has been recognized as a synonym of *Kaburakia excelsa* Bock, 1925 (Kato 1937). Faubel (1983) reassigned *C. aegypticus*, *C. eximius*, and *C. bartschi* to the family Callioplanidae Hyman, 1953, a family characterized by a smooth or somewhat wavy inner epithelium of the prostatic vesicle. While the remaining two species, *C. wahlbergi* and *C. sondaicus*, had been placed in the family Pseudostylochidae Faubel, 1983, in which the inner epithelium of the prostatic vesicle is tubularly chambered. Within Callioplanidae, he further assigned *C. aegypticus* and *C. eximius* to the genus *Trigonoporus* Lang, 1884, with the ductus vaginalis opening to the exterior well behind the female pore, and assigned *C. bartschi* to the genus *Tokiphallus* Faubel, 1983, based on the male apparatus being enclosed in a common muscular bulb. However, our review of the original descriptions shows that Faubel’s statement of the ductus vaginalis was incorrect. In fact, in the original descriptions of *C. aegypticus* and *C. eximius*, the authors clearly stated that the ductus vaginalis and vagina externa open into a common pore, which was also illustrated in the schematic figures of the copulatory apparatus of *C. aegypticus* (Melouk 1940: fig. 5) and *C. eximius* (Kato 1937: fig. 11). In addition, *C. wahlbergi* and *C. sondaicus* were originally described as having small folds in the proximal part of the prostatic vesicle (see Bock 1913, 1925), and thus do not possess a “tubularly chambered” epithelium. Therefore, Faubel’s classification of *C. wahlbergi* and *C. sondaicus* appears to be inaccurate. Taken together, we suggest that *C. aegypticus*, *C. eximius*, *C. wahlbergi* and *C. sondaicus* should be returned to *Cryptophallus*. Although *C. bartschi* differs from *Cryptophallus* in having the male apparatus enclosed in a common muscular bulb and the male copulatory organ positioned slightly posterior to the pharyngeal cavity (Kaburaki 1923), whether these characteristics are sufficient to classify it as a different genus (*Tokiphallus*) still requires further investigation.

#### Cryptophallus sinensis

Taxon classificationAnimaliaPolycladidaPseudostylochidae

Liu sp. nov.

6ECB32CB-53A5-5312-9EFE-67B9D6151956

https://zoobank.org/C0A65789-DADB-430D-84FF-8D50849AC9EE

Figs 1, 2

##### Etymology.

The specific name is an adjective derived from the Latin *sinensis*, meaning “Chinese”, referring to the species being found in Chinese waters.

##### Material examined.

***Holotype***:China • hermaphrodite; Guangdong Province, Huidong; 22°44.95'N, 114°45.05'E; 15 Apr. 2025; Hai-Long Liu leg.; sagittal sections on 8 slides; GenBank accession: PZ111956 (COI), PZ111958 (16S), PZ112129 (18S) and PZ112134 (28S); MBM288501.***Paratypes***:China • hermaphrodite; same data as for holotype; sagittal sections on 7 slides; GenBank accession: PZ111959 (16S), PZ112130 (18S) and PZ112135 (28S); MBM288502.

##### Description.

**Figure 1.**
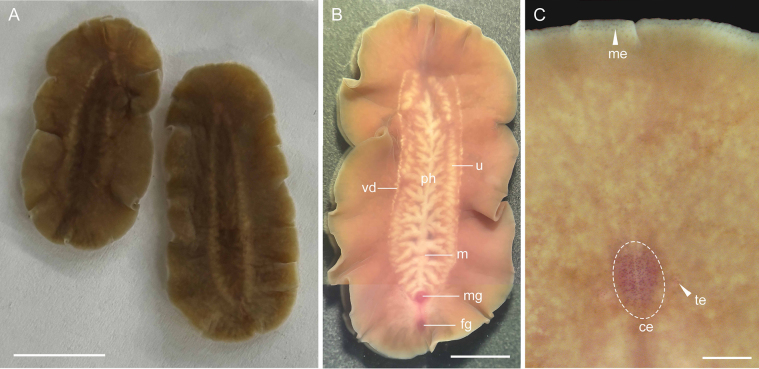
*Cryptophallus sinensis* sp. nov., photographs taken in anesthetized specimens. **A**. Dorsal view; **B**. Ventral view; **C**. Eyespots arrangement (white arrows indicating tentacular eyespot and marginal eyespot; dashed ellipse showing cerebral eyespot). Abbreviations: ce = cerebral eyespot, fg = female gonopore, m = mouth, me = marginal eyespot, mg = male gonopore, ph = pharynx, te = tentacular eyespot, u = uteri, vd = vasa deferntia. Scale bars: 10 mm (**A**); 5 mm (**B**); 1 mm (**C**).

Body is elongate oval, both ends are round (Fig. 1A, B). Anesthetized specimens are 31–25 mm in length and 15 mm in width. Dorsal surface is grayish brown, slightly darker in the median (Fig. 1A), and the ventral paler. The extreme marginal of body is colorless. Brain and genital regions are reddish. A pair of small knob-like nuchal tentacles are located 6 mm from the anterior end and 1.7 mm apart from each other. Each has 5–8 tentacular eyespots. Numerous cerebral eyespots form a crowded cluster, within which two groups can be faintly distinguished (Fig. 1C). Frontal eyespots are scattered over the anterior end of the body (Fig. 1C). Marginal eyespots are distributed around the entire body.

A ruffled pharynx is located at the center of the body, measuring 18–22 mm in length (Fig. 1B). The mouth is located in the posterior region of the pharynx, approximately 5 mm from the posterior end of the pharynx (Fig. 1B). The male pore is 6 mm from the posterior end of the body, and the female pore opens 2.5 mm behind the male pore (Fig. 1B).

**Figure 2.**
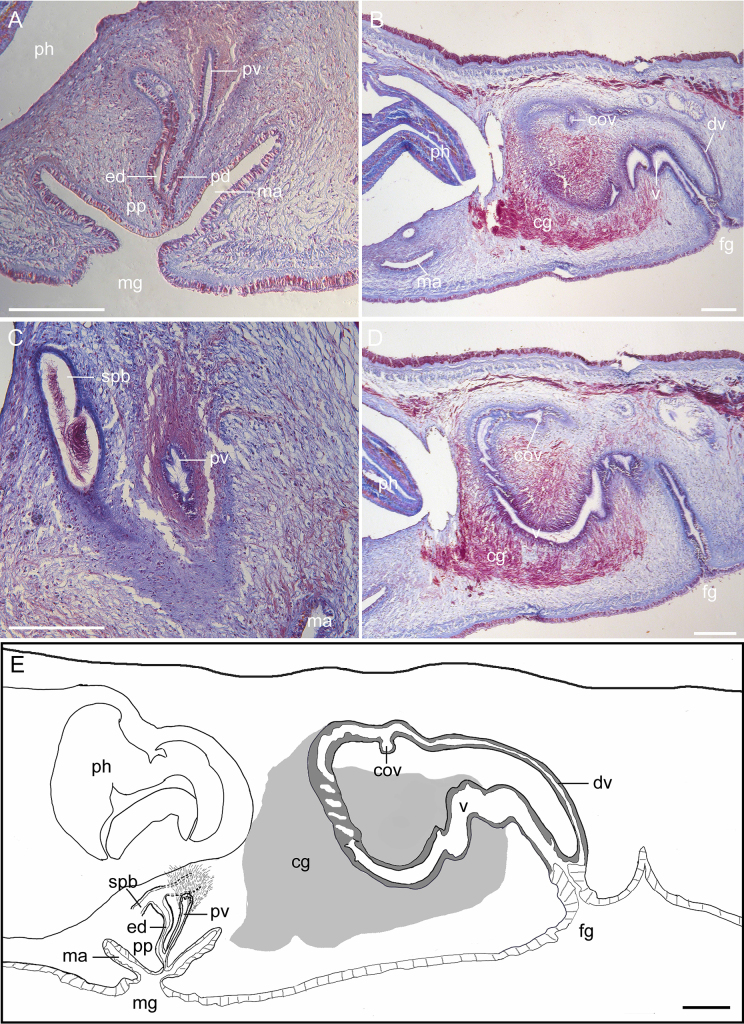
*Cryptophallus sinensis* sp. nov., sagittal sections (**A–D**) and schematic diagram (**E**). **A**. Male copulatory apparatus; **B**. Female copulatory apparatus; **C**. Prostatic vesicle; **D**. Vagina; **E**. Schematic diagram of copulatory complex. Abbreviations: cg = cement glands, cov = common oviduct, dv = ductus vaginalis, ed = ejaculatory duct, fg = female gonopore, ma = male atrium, mg = male gonopore, pd = prostatic duct, ph = pharynx, pp = penis papilla, pv = prostatic vesicle, spb = spermiducal bulbs, v = vagina. Scale bars: 200 µm (**A–E**).

The male copulatory apparatus is not enclosed by a muscular bulb and is comprised of the spermiducal bulbs, a free prostatic vesicle, and an unarmed penis papilla. A pair of vasa deferntia run ventrally along the outer sides of the uteri, and extend anteriorly to the region posterior to the brain (Fig. 1B). With the musculature slightly increasing in thickness in the distal part, they develop into elongate tubular spermiducal bulbs that are difficult to distinguish from the vasa deferntia as separate organs. They converge near the proximal end of the prostatic vesicle to form an ejaculatory duct. The prostatic vesicle is very small and club-shaped, located beneath the posterior end of the pharynx, and positioned nearly vertically to the base of the penis (Fig. 2A, E). The prostatic vesicle is lined with a simple ciliated epithelium and bears small epithelial folds at its proximal end (Fig. 2C). The prostatic duct joins the ejaculatory duct near the tip of the penis papilla (Fig. 2A, E). A wide, conical penis projects vertically into the male atrium, which is lined with ciliated epithelium (Fig. 2A, E).

Ovaries are situated dorsally. A pair of coiled uteri commence from the level of anterior end of the pharynx and run posteriorly along both sides of the pharynx (Fig. 1B). The female copulatory apparatus is generally similar to that of other species in the genus, comprising a ductus vaginalis that lacks an outer opening of its own and opens together with the vagina into a common antrum (Fig. 2B, E). The vagina extends upward from the female genital pore for a moderate distance, then runs anteriorly parallel to the ventral side of the body, curves sharply upwards and backwards, and after a short distance unite in a common oviduct (Fig. 2B, D). Beyond this point, the ductus vaginalis loops back towards the female antrum (Fig. 2B, E). In the sharply upward-curving portion of the vagina, it is twisted into a distinct spiral coil, making approximately five complete turns (Fig. 2D). Cement glands are stained with acid fuchsin, empty into the portions of the vagina that run parallel to the ventral side of the body and that curve sharply upward (Fig. 2B, D, E).

##### Distribution.

Huidong, Guangdong, China; Singapore.

##### Habitat.

Intertidal, under the stone.

##### Molecular phylogeny.

*Cryptophallus sinensis* sp. nov. is sister to a clade composed of *Leptostylochus cf. gracilis* and *Leptostylochus victoriensis* with poor support (51/0.54) and nested within Stylochoidea (Fig. 5; Suppl. material 1: fig. S1).

##### Remarks.

As aforementioned, the revised genus currently comprises five species. *Cryptophallus sinensis* sp. nov. can be distinguished from its four congeners (except *C. japonicus*) by having a grayish-brown dorsal pattern with darker in the median region (Table 1). Additionally, the new species has fewer tentacular eyespots (5–8) than those in *C. eximius*, *C. sondaicus*, and *C. wahlbergi* (all > 13, Table 1). In *C. aegypticus* and *C. sondaicus*, the ejaculatory duct and prostatic duct open separately at the distal end of the penis, whereas in *C. sinensis* sp. nov. the ejaculatory duct connects distally to the prostatic duct (Table 1). Furthermore, *C. eximius* has the mouth opening in the anterior part of the body; *C. wahlbergi* possesses a prostatic vesicle with a proximally enlarged lumen (Bock 1913: textfig. 13), and *C. sondaicus* presents a rudimentary duct connected with the ductus vaginalis, which further distinguish these congeners from *C. sinensis* sp. nov. (Table 1). *Cryptophallus sinensis* sp. nov. differs from *C. japonicus* by its body color (grayish-brown dorsal pattern vs. light silvery black) and the arrangement of the cerebral eyespots (two scarcely separate clusters vs. a single cluster) (Table 1). In addition, the middle portion of the vagina forms a distinct spiral coil with approximately five complete turns in the new species, while in *C. japonicus* it is raised to nine (Kato 1938: fig. 6).

**Table 1.** Comparison of morphological characters between species of *Cryptophallus*.

Species	*C. aegypticus*	*C. eximius*	*C. sondaicus*	*C. wahlbergi*	*C. sinensis* sp. nov.	*C. japonicus*
Body size (mm)	20–65 × 12–35; 62 (mature)	50 × 30 (preserved state)	40 × 20.5	40 × 21	31–25 × 15 (anesthetized state)	40 × 20
Color pattern	uniformly light creamy	uniformly grayish brown	uniformly dark grey- brown without concentration of the median line	uniformly dark brown	grayish brown, darker in the median	Light silvery black, darker in the median
Cerebral eyespots	two scarcely separate clusters	a single cluster	two scarcely separate clusters	a single cluster	two scarcely separate clusters	a single cluster
Tentacular eyespots	6–8, underneath tentacular knobs	about 13	about 20, underneath tentacular knobs	about 13 (Textfig. 12a) underneath tentacular knobs	5–8, within the tentacular knobs	4–5, underneath tentacular knobs
Mouth	near posterior third of body	anterior part of the body	near posterior fourth of body	near posterior fourth of body	near posterior fourth of body	near posterior third of body
Male and female reproductive anatomy	pv: small, lined with smooth epithelium; ejaculatory duct and prostatic duct open separately at the distal end of penis; rudimentary duct connected with the ductus vaginalis absent	pv: small, lined with smooth epithelium; ejaculatory duct connects to prostatic duct at the distal end of penis; rudimentary duct connected with the ductus vaginalis absent	pv: small, lined with small epithelial folds at its proximal end; ejaculatory duct and prostatic duct open separately at the distal end of penis; rudimentary duct connected with the ductus vaginalis present	pv: relatively large, with a proximally enlarged lumen and lined with small epithelial folds; ejaculatory duct connects to prostatic duct at the distal end of penis; rudimentary duct connected with the ductus vaginalis absent	pv: small, lined with small epithelial folds at its proximal end; ejaculatory duct connects to prostatic duct at the distal end of penis; rudimentary duct connected with the ductus vaginalis absent	pv: small, lined with smooth epithelium; ejaculatory duct connects to prostatic duct at the distal end of penis; rudimentary duct connected with the ductus vaginalis absent
Type locality	Gulf of Suez	Japan	Amboina	South Africa	China	Japan
Reference	Melouk 1940	Kato 1937	Bock 1925	Bock 1913	This study	Kato 1938, 1944

The 28S rDNA sequence of *C. sinensis* sp. nov. shows high similarity with that of Acotylea 9 (ZRC.PLA.0269, ZRC.PLA.0273) collected from Singapore (Ong and Tong 2018; Diong et al. 2025), with BLAST identities of 99.89% (PQ863117) and 99.79% (PQ863131), respectively. By contrast, the sequence identity between the two Singaporean Acotylea 9 specimens was 99.39%. This level of intraspecific sequence variation, together with the matching color pattern (see fig. 16A, B in Ong and Tong 2018) suggest that these specimens may be conspecific. Nevertheless, we acknowledge that this hypothesis should be further tested using additional molecular markers and detailed comparisons of internal morphology, due to the limited ability of 28S rDNA to distinguish species (Oya and Kajihara 2017).

###### Family Limnostylochidae Faubel, 1983

**Genus *Limnoplana* Faubel, 1983**

#### Limnoplana obscuriviridis

Taxon classificationAnimaliaPolycladidaLimnostylochidae

Liu sp. nov.

1890092E-7799-5F07-8F75-F4135CC93F3F

https://zoobank.org/9AD7BF66-D7D6-498B-BECC-0547F72F205B

Figs 3, 4

##### Etymology.

The specific name is a compound of the Latin adjective *obscure* (dark) and virid (green), referring to the dark-green dorsal coloration of the body.

##### Material examined.

***Holotype***:China • hermaphrodite; Guangdong Province, Shenzhen; 22°31.33'N, 113°57.06'E; 6 Dec. 2024; Bing-Bing He leg.; underneath stones in the man­groves; sagittal sections on 19 slides; GenBank accession: PZ111957 (COI), PZ111960 (16S), PZ112131 (18S) and PZ112136 (28S); MBM288503.***Paratypes***:China • 1 hermaphrodite; same data as for holotype; sag­ittal sections on 18 slides; GenBank accession: PZ111961 (16S), PZ112132 (18S) and PZ112137 (28S); MBM288504 • 1 hermaphrodite; same data as for holotype; sag­ittal sections on 18 slides; GenBank accession: PZ111962 (16S), PZ112133 (18S) and PZ112138 (28S); MBM288505.

##### Description.

**Figure 3.**
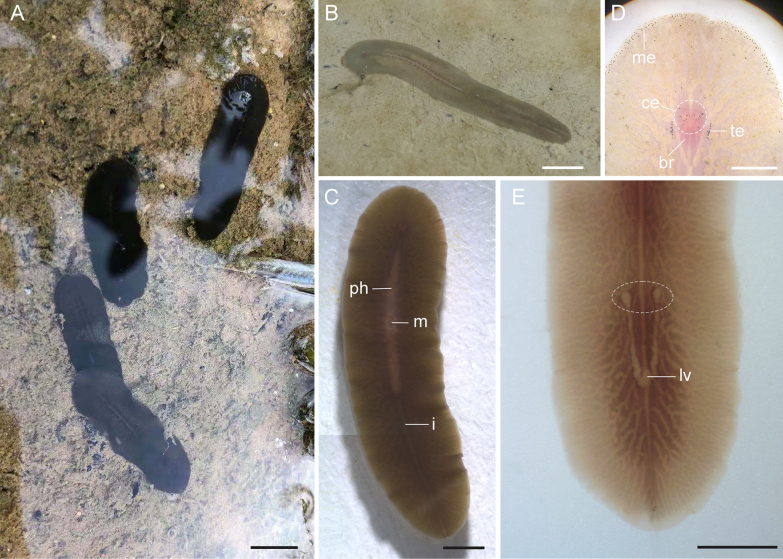
*Limnoplana obscuriviridis* sp. nov., photographs taken in life from habitat (**A, B**) and anesthetized specimens (**C–E**). **A, B**. Dorsal view; **C**. Ventral view; **D**. Head region (dashed ellipse showing cerebral eyespot); **E**. Posterior body region (dashed ellipse showing the two swollen ends of Lang’s vesicle). Abbreviations: br = brain, ce = cerebral eyespot, i = intestine, lv = Lang’s vesicle, m = mouth, me = marginal eyespot, ph = pharynx, te = tentacular eyespot. Scale bars: 5 mm (**A**); 2 mm (**B, C, E**); 1 mm (**D**).

The body is thin and elongate oval, with the posterior end slightly narrowed (Fig. 3A). Anesthetized specimens are 16–35 mm long and 4–7 mm wide, while mature adults are approximately 30 mm long and 7 mm wide. The dorsal surface is dark green or khaki greenish, with two darker stripes running along the midline (Fig. 3A, B). The ventral surface is lighter than the dorsal. Some of the smaller specimens (approximately 12 mm long and 4 mm wide) are somewhat reddish. The extreme margin of the body is colorless, and while the heart-shaped brain region is reddish (Fig. 3B, D). Tentacles are absent. Tentacular eyespots are arranged in two long clusters, each consisting of 18–35 eyespots (Fig. 3D). Cerebral eyespots are scattered between tentacular eyespots, totaling approximately 30 eyespots (Fig. 3D). Marginal eyespots encircle the entire body, with more than one layers in the anterior 1/4 of the body (Fig. 3D), while only one sparse layer in the remaining regions of the body.

**Figure 4.**
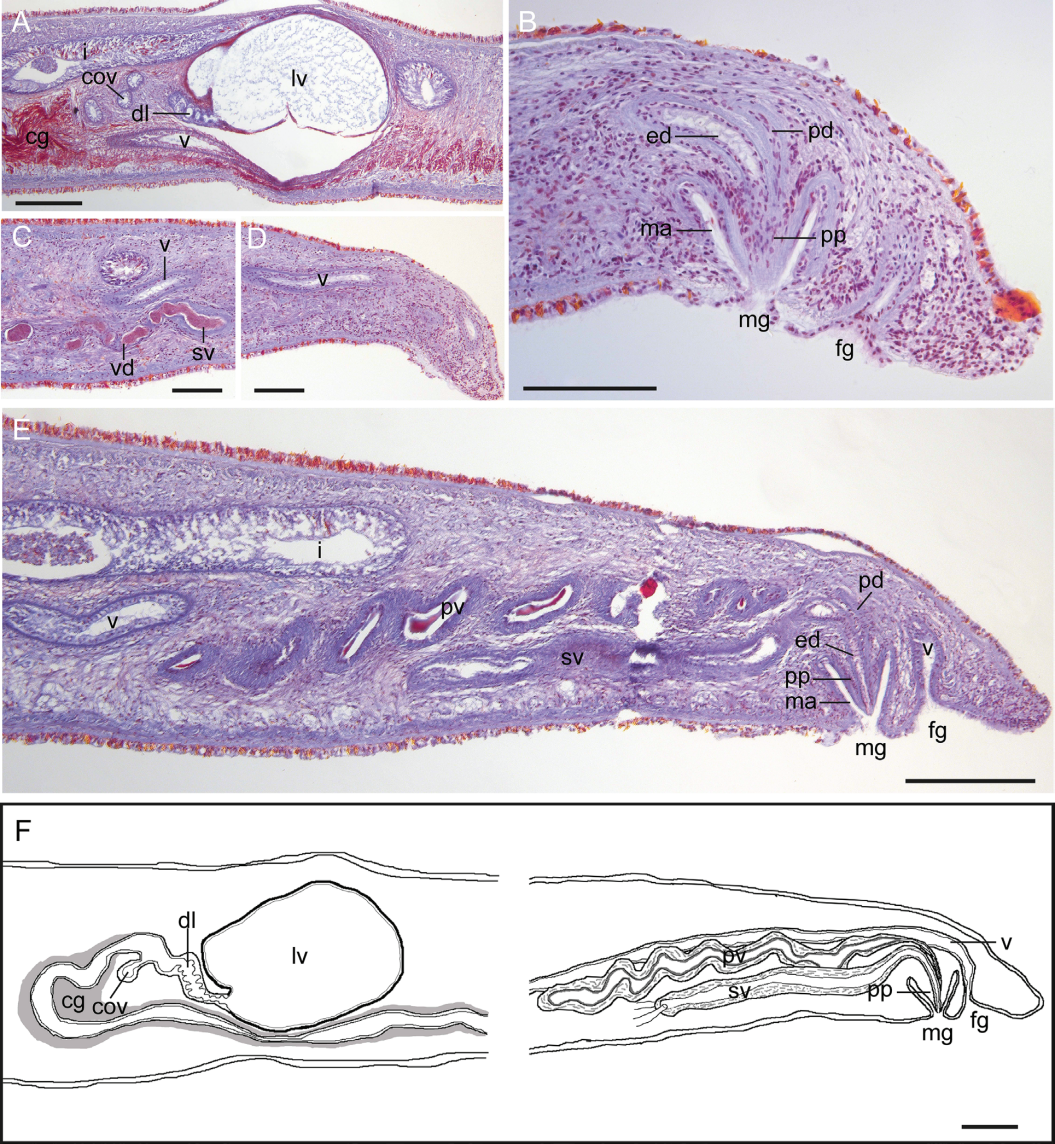
*Limnoplana obscuriviridis* sp. nov., sagittal sections (**A–E**) and schematic diagram (**F**). **A, D**. Female copulatory apparatus; **B, C, E**. Male copulatory apparatus; **F**. Schematic diagram of copulatory complex. Abbreviations: cg = cement glands, cov = common oviduct, dl = duct of Lang’s vesicle, ed = ejaculatory duct, fg = female gonopore, i = intestine, lv = Lang’s vesicle, ma = male atrium, mg = male gonopore, pd = prostatic duct, pp = penis papilla, pv = prostatic vesicle, sv = seminal vesicle, v = vagina, vd = vasa deferntia. Scale bars: 200 µm (**A**, **E**, **F**); 100 µm **(B–D**).

A ruffled pharynx is located in the anterior half of the body, measuring 6–10 mm in length (Fig. 3C). The mouth is located in the middle region of the pharynx, approximately at the anterior one-third of the body. (Fig. 3C). Genital pores are situated at the posterior end of the body: the male pore is located approximately 0.3 mm from the posterior end, and the female pore opens about 0.1 mm behind the male pore (Fig. 4B, E).

The male copulatory apparatus is comprised of a true seminal vesicle, a free prostatic vesicle, and an unarmed penis. A pair of vasa deferntia run ventrally, enter the proximal end of the elongate tubular seminal vesicle separately (Fig. 4C, F). The seminal vesicle (0.5–0.7 mm in its long axis) has a distinct muscular wall, and is situated beneath the prostatic vesicle (Fig. 4E). Its distal end gradually narrows to form the ejaculatory duct, which runs obliquely backward and upward for a short distance, then bends sharply before opening at the tip of the penis (Fig. 4E, F). The prostatic vesicle (0.7–1 mm in its long axis) is an elongate tube running in a wavy manner, with a strong muscular wall and is lined with smooth epithelium (Fig. 4E, F). The prostatic secretion is strongly stained by acid fuchsin, taking on a somewhat dirty red hue (Fig. 4E). The prostatic duct receives the ejaculatory duct at the base of penis (Fig. 4B). A conical penis without stylet projects vertically into the male atrium (Fig. 4B, E, F).

The ovaries are situated dorsally. The distal ends of a pair of uteri fuse at the midline to form a common oviduct, which runs upward to enter the vagina (Fig. 4A, F). From this point, Lang’s duct with numerous radial folds runs postero-ventrally, and leads to Lang’s vesicle (Fig. 4A, F). Lang’s vesicle is “U” shaped, with both ends swollen into a spherical form (Fig. 3E), and lined with a thin columnar epithelium. Lang’s vesicle contains granular secretion that stain blue with aniline blue (Fig. 4A). The vagina extends anterior for a short distance, then curves postero-ventrally, running along ventral side to near the proximal end of the prostatic vesicle; then vagina runs obliquely upward and backward, extending for some distance close to the dorsal side, eventually turning ventrally to open at the female gonopore (Fig. 4A, D, E, F). The vagina is lined with both ciliated and smooth epithelium. From the common oviduct to the proximal end of the prostatic vesicle, the vagina is surrounded by cement glands, which are stained with acid fuchsin (Fig. 4A, F).

##### Distribution.

The species is known from Shenzhen, Guangdong, China.

##### Habitat.

The specimens were collected from the estuary of the Dasha River, where the salinity is approximately 4‰ and mangroves are present. They were found intertidally underneath stones in the mangroves.

##### Molecular phylogeny.

**Figure 5.**
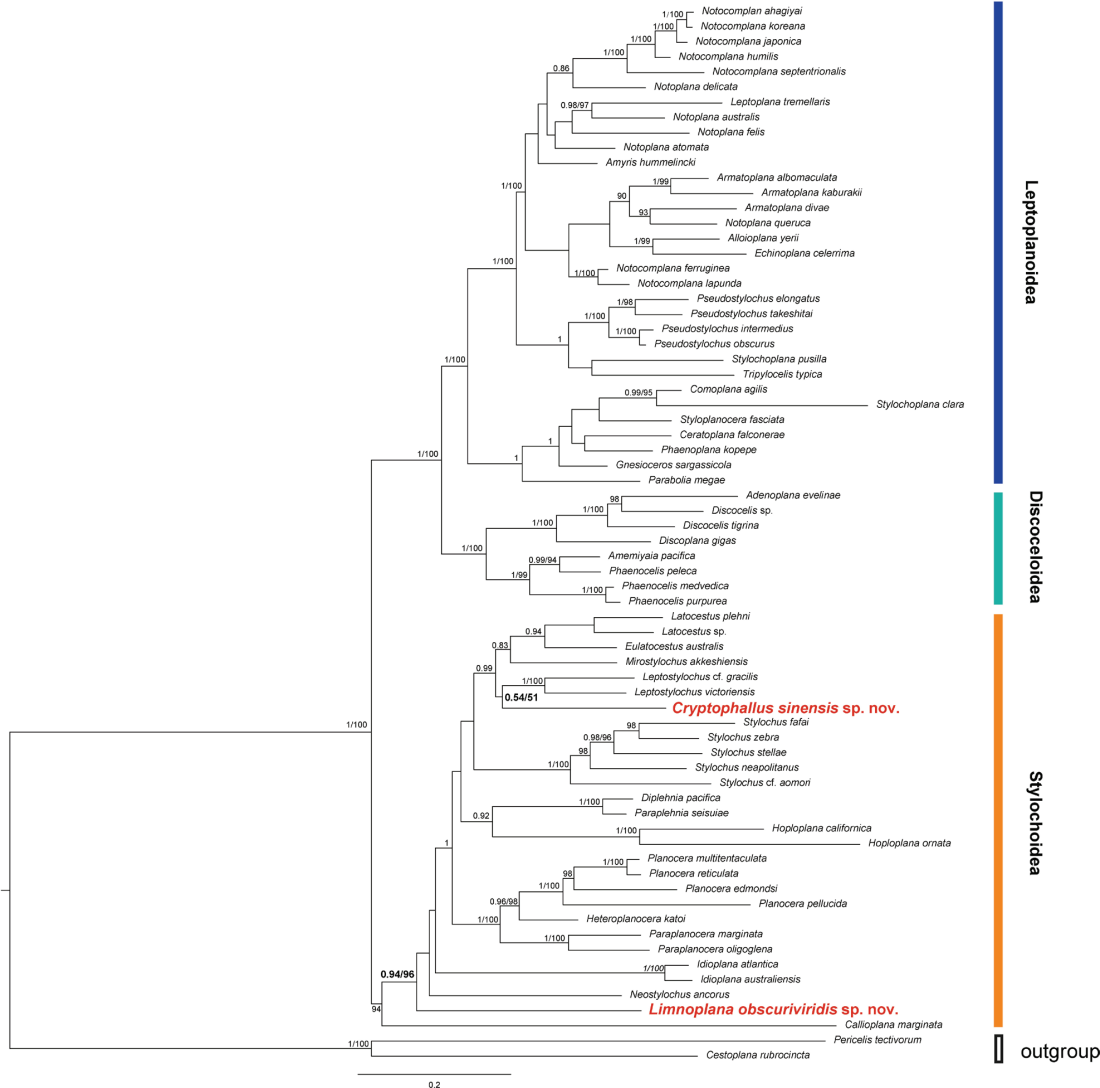
Bayesian phylogenetic tree based on a concatenated dataset of partial 18S, 28S, 16S and COI sequences. Numbers near nodes are posterior probability (PP ≥ 0.8) and bootstrap value (BS ≥ 90%), respectively.

*Limnoplana obscuriviridis* sp. nov was positioned inside the Stylochoidea superfamily with high support (96/0.94), clustering with all other stylochoids except *Callioplana marginata* (Fig. 5; Suppl. material 1: fig. S1).

##### Remarks.

There are two recognized species in this genus: *Limnoplana annardalei* (Kaburaki, 1918) and *Limnoplana amara* (Kaburaki, 1918), which were described from Koh Yaw Island, Talé Sap and Singgora, Thailand, respectively. *Limnoplana obscuriviridis* sp. nov. can be distinguished from two congeners by three key morphological characters: i) presence of a true seminal vesicle (absent in *L. annardalei* and *L. amara*); ii) the two swollen ends of Lang’s vesicle (not illustrated as swollen in *L. annardalei*; Kaburaki 1918: plate VIII, fig. 1; *L. amara* described as similar to *L. annardalei*; this character was possibly overlooked or not explicitly described by Kaburaki); and iii) a distinct dorsal pattern characterized by two dark longitudinal stripes along the midline (uniform olive-greenish without any marking in *L. annardalei* and *L. amara*) (Kaburaki 1918). In addition, the new species differs from *L. annardalei* by the position of the genital organ (the male pore is situated at the posterior tenth of the body, about 1 mm from the posterior end in *L. annardalei*; while in *L. obscuriviridis* sp. nov, it is closer to the posterior end of the body, approximately 0.3 mm from the posterior end); and the shape of the brain (kidney-shaped in *L. annardalei* vs. heart- shaped in *L. obscuriviridis* sp. nov.) (Kaburaki 1918). Notably, the tentacular and cerebral eyespots blend together in *L. amara*, which distinguishes it from *L. obscuriviridis* sp. nov. (Kaburaki 1918).

## Discussion

Species of the genera *Cryptophallus* and *Limnoplana* were described approximately a century ago, yet they lack high-quality live photographs, detailed images of the reproductive structures, and molecular data. Accordingly, their taxonomy has received little revision since then. In this study, we describe two new species in these long-neglected genera by integrating morphological and molecular evidence. We assessed the phylogenetic positions of the two aforementioned genera, represented by the newly described species, among other acotylean polyclads by molecular phylogenetic analyses using concatenated COI, 16S, 18S, and 28S sequences.

We revise Faubel’s (1983) classification of the genus *Cryptophallus*. According to Faubel (1983), the inner epithelium of the prostatic vesicle in *Cryptophallus* is “tubularly chambered”, which should be classified into Pseudostylochidae. This assessment of a “tubularly chambered” prostatic vesicle lining in *C. wahlbergi* by Faubel (1983) was presumably based on an illustration from Bock (1913: textfig. 13). In fact, Bock’s illustration is more similar to our Fig. 2C, in which the prostatic vesicle bears epithelial folds at its proximal end rather than exhibiting tubularly chambered structure. In addition, the inner lining of the prostatic vesicle in *C. sinensis* sp. nov. is unstable and varies across developmental stages. For example, in the more mature specimen (MBM288501; larger in size and with more fully developed eggs), the prostatic vesicle bears epithelial folds at its proximal end (Fig. 2C); in contrast, such epithelial folds are absent in the less mature specimen (MBM288502) (Fig. 2A). These findings indicate that the epithelial lining of the prostatic vesicle is not a reliable diagnostic character for *Cryptophallus*, as this trait may be influenced by ontogenetic maturity, secretory state, and fixation artifacts. In contrast, we consider that the size and position of the prostatic vesicle may provide more reliable generic diagnostic characters. In the female copulatory apparatus, all *Cryptophallus* species possess a ductus vaginalis that opens together with the vagina into a common antrum, which represents a key diagnostic character of the genus. Additionally, a rudimentary duct connected to the ductus vaginalis has so far been reported only in *C. sondaicus*. Therefore, we suggest that this feature should be interpreted with caution in distinguishing species until additional histological material is available from more species of the genus.

Although the familial affiliation of *Cryptophallus* remains unresolved, our study shows that *Cryptophallus* does not belong to Pseudostylochidae (Faubel 1983) or Callioplanidae (Faubel 1983). In our phylogenetic trees, *C. sinensis* sp. nov. is sister to a clade comprising two *Leptostylochus* species, and both cluster with the family Latocestidae. *Leptostylochus* was originally assigned to Stylochidae, but molecular phylogenetic studies (Oya and Kajihara 2020; Rodríguez et al. 2021), which align with our results, do not support this affiliation. *L. victoriensis*, *L. cf. gracilis* and *C. sinensis* sp. nov. share the following morphological characters with latocestids: i) presence of marginal eyespots, ii) male copulatory apparatus with papilla-like unarmed penis and predominantly with spermiducal bulbs, and iii) mouth opening at the posterior half of the pharynx. Currently, the familial affiliations of both *Leptostylochus* and *Cryptophallus* require re-evaluation through the inclusion of more species from both genera.

The family Limnostylochidae is divided into two genera, which are primarily distinguished by the presence/absence of a penis stylet and a seminal vesicle: *Limnostylochus* possesses a penis stylet and a seminal vesicle, whereas *Limnoplana* possesses an unarmed penis papilla and lacks a seminal vesicle (Faubel 1983). Here, we classify our new species into *Limnoplana*, although it possesses a true seminal vesicle. We place greater taxonomic emphasis on the armature of the penis than on more subjective traits of the seminal vesicle. Kaburaki (1918) originally described *L. annardalei* as follows: “The seminal canals (sc), running backwards along the sides of the hind parts of the main gut, somewhat widen in part and thus serve as accessory seminal vesicles. Posteriorly they gradually narrow and unite into an unpaired median duct, the ejaculatory duct (ed), at a point far in front of the male aperture (o). A true seminal vesicle does not exist.” In contrast, in *L. obscuriviridis* sp. nov., a pair of vasa deferntia run ventrally and enter an elongate tubular seminal vesicle; the latter with a distinct muscular wall that can be easily distinguished from the vasa deferntia. We consider that whether the aforementioned ejaculatory duct exhibits a thickened muscular wall forming a tubular seminal vesicle may be affected by fixation and age of the specimens. Clearly, in Limnostylochidae, “presence/absence of a penis stylet” represents a character of greater taxonomic value than plastic traits such as the seminal vesicle. Accordingly, we propose minor revisions to the generic diagnosis of *Limnoplana* as follows: *Limnoplana* without tentacles; marginal eyes encircling the entire body margin; prostatic vesicle exceedingly elongated running in wave manner; seminal vesicle and spermiducal bulbs absent or elongate seminal vesicle present; penis papilla unarmed; the female apparatus directed backwards and running dorsally along the male complex; and U-shaped Lang’s vesicle present. Since 1918, *L. obscuriviridis* sp. nov. has been the first newly described species of *Limnoplana*. Additionally, we provide the first molecular makers for Limnostylochidae, and our molecular phylogenetic analyses indicate that this family belongs to the superfamily Stylochoidea.

All species of the family Limnostylochidae inhabit freshwater or brackish water (Stummer-Traunfels 1902; Kaburaki 1918), suggesting that tolerance to low salinity may represent a conserved ecological trait within this lineage. Our molecular analyses showed that Limnostylochidae belongs to the superfamily Stylochoidea. This placement is further supported by the presence of a free prostatic vesicle, which is a diagnostic feature of Stylochoidea. Limnostylochids differ from other stylochoids in possessing an elongated, wavy prostatic vesicle, which may be associated with ecological adaptation, although this hypothesis requires further comparative study.

## Supplementary Material

XML Treatment for Cryptophallus

XML Treatment for Cryptophallus sinensis

XML Treatment for Limnoplana obscuriviridis

## Additional information

### Use of AI

No use of AI was reported.

### Author ORCIDs

Hai-Long Liu https://orcid.org/0009-0008-4677-0568

Bing-Bing He https://orcid.org/0009-0008-9323-6050

Shuang-Fei Li https://orcid.org/0000-0002-5045-9165

An-Tai Wang https://orcid.org/0000-0003-4222-9535

Zhang-Li Hu https://orcid.org/0000-0002-2596-8515

Yu Zhang https://orcid.org/0000-0001-7378-6946

### Data availability

All of the data that support the findings of this study are available in the main text or Supplementary Information.

## Supplementary materials

Supplementary material 1 Suplementary tables and figures Data type docx Explanation note **table SS1**. List of primers used in this study. **table S2**. List of species included in the molecular phylogenetic analyses. **figure S1**. Maximum-likelihood tree based on a concatenated dataset of partial 18S, 28S, 16S and COI sequences. https://binary.pensoft.net/file/1573652This dataset is made available under the Open Database License (http://opendatacommons.org/licenses/odbl/1.0/). The Open Database License (ODbL) is a license agreement intended to allow users to freely share, modify, and use this Dataset while maintaining this same freedom for others, provided that the original source and author(s) are credited. Hai-Long Liu, Bing-Bing He, Shuang-Fei Li, An-Tai Wang, Zhang-Li Hu, Yu Zhang
