# Supplementary material for: Morphology and phylogeny of two acotylean flatworms (Platyhelminthes, Polycladida) from the genera Cryptophallus and Limnoplana in the South China Sea
Source: Zookeys. 2026 Mar 30;1275:211–27. doi: 10.3897/zookeys.1275.180665 (PMC13054336; doi:10.3897/zookeys.1275.180665)
Supplement: Supplementary material 1 — Suplementary tables and figures [file zookeys-1275-211_article-180665__-s001.docx]

| Gene | Primer name | Sequence | Reference |
| --- | --- | --- | --- |
| 18S | hrms18S_F | ATCCTGCCAGTAGTCATATGC | Oya and Kajihara 2020 |
|  | hrms18S_R | CTACGGAAACCTTGTTACGAC |  |
| 28S | fw1 | AGCGGAGGAAAAGAAACTA | Sonnenberg et al. 2007 |
|  | rev2 | ACGATCGATTTGCACGTCAG |  |
| COI | Acotylea_COI_F | ACTTTATTCTACTAATCATAAGGATATAGG | Oya and Kajihara 2017 |
|  | Acotylea_COI_R | CTTTCCTCTATAAAATGTTACTATTTGAGA |  |
| 16S | 16SarL | CGCCGTTTATCAAAAACAT | Palumbi et al. 1991 |
|  | 16SbrH | CCGGTCTGAACTCAGATCACGT |  |

**Table S1** List of primers used in this study.

**References**

Oya Y, Kajihara H (2017) Description of a new *Notocomplana* species (Platyhelminthes: Acotylea), new combination and new records of Polycladida from the northeastern Sea of Japan with a comparison of two different barcoding markers. Zootaxa 4282(3): 526–542. https://doi.org/10.11646/zootaxa.4282.3.6

Oya Y, Kajihara H (2020) Molecular phylogenetic analysis of Acotylea (Platyhelminthes: Polycladida). Zoological Science 37(3): 271–279. https://doi.org/10.2108/zs190136

Palumbi S, Martin A, Romano S, McMillan WO, Stice L, Grabowski G (1991) The Simple Fools Guide to PCR, Ver. 2. Department of Zoology and Kewalo Marine Laboratory, University of Hawaii, Honolulu

Sonnenberg R, Nolte AW, Tautz D (2007) An evaluation of LSU rDNA D1–D2 sequences for their use in species identification. Frontiers in Zoology 4(1): 1–12. https://doi.org/10.1186/1742-9994-4-6

| **Table S2** List of species included in the molecular phylogenetic analyses. | | | | | | | |
| --- | --- | --- | --- | --- | --- | --- | --- |
| Family | Species | 18S | 28S | 16S | COI | Locality | References |
| **Discoceloidea** |  |  |  |  |  |  |  |
| Cryptocelidae | *Amemiyaia pacifica* Kato, 1944 | LC508166 | LC508143 | LC508185 | LC508203 | Wakayama, Japan | Oya & Kajihara (2020) |
|  | *Phaenocelis medvedica* Marcus, 1952 | NA | KY263706 | NA | NA | Catuama, Pernambuco State, Brazil | Bahia *et al*. (2017) |
|  | *Phaenocelis peleca* Marcus & Marcus, 1968 | NA | MH700342 | NA | NA | Santa Marta, Colombia | Litvaitis *et al*. (2019) |
|  | *Phaenocelis purpurea* (Schmarda, 1859) | NA | MH700346 | NA | NA | South Water Caye, Belize | Litvaitis *et al*. (2019) |
| Ilyplanidae | *Discoplana gigas* (Schmarda, 1859) | LC508162 | LC508139 | NA | LC508199 | Okinawa, Japan | Oya & Kajihara (2020) |
| Discocelidae | *Discocelis tigrina* (Blanchard, 1847) | MN334200 | MN384690 | NA | NA | Valencia, Spain | Dittmann *et al*. (2019) |
|  | *Discocelis* sp. | LC508170 | LC508146 | LC508189 | LC508206 | Kumamoto, Japan | Oya & Kajihara (2020) |
|  | *Adenoplana evelinae* Marcus, 1950 | NA | MH700268 | NA | NA | Buttonwood, Isla Solarte Bocas del Toro, Panama | Litvaitis *et al*. (2019) |
| **Leptoplanoidea** |  |  |  |  |  |  |  |
| Gnesiocerotidae | *Echinoplana celerrima* Haswell, 1907 | MW376754 | MW377507 | MW376599 | MW375911 | New South Wales, Australia | Rodríguez *et al*. (2021) |
|  | *Gnesioceros sargassicola* (Mertens, 1833) | NA | MH700309 | NA | NA | Santa Marta, Colombia | Litvaitis *et al*. (2019) |
|  | *Styloplanocera fasciata* (Schmarda, 1859) | NA | MH700408 | NA | NA | Pos Spaño, Curaçao | Litvaitis *et al*. (2019) |
|  | *Ceratoplana falconerae* Rodríguez et al., 2021 | MW376740 | MW377493 | MW376585 | MW375897 | Victoria, Australia | Rodríguez *et al*. (2021) |
|  | *Parabolia megae* Rodríguez et al., 2021 | MW376744 | MW377497 | MW376589 | MW375901 | New South Wales, Australia | Rodríguez *et al*. (2021) |
| Leptoplanidae | *Leptoplana tremellaris* (Müller, 1773) | MN421937 | MN421931 | NA | NA | Cornwall, UK | Dittmann *et al*. (2019) |
| Notocomplanidae | *Notocomplana ferruginea* (Schmarda, 1859) | NA | MH700324 | NA | NA | Peanut Island, Florida, USA | Litvaitis *et al*. (2019) |
|  | *Notocomplana hagiyai* Oya & Kajihara, 2017 | LC508152 | LC508129 | LC176041 | LC176003 | Hokkaido, Japan | Oya & Kajihara, (2017, 2020) |
|  | *Notocomplana humilis* (Stimpson, 1857) | LC508168 | LC508144 | LC508187 | LC508204 | Wakayama, Japan | Oya & Kajihara (2020) |
|  | *Notocomplana japonica* (Kato, 1937) | LC508154 | LC508131 | LC176051 | LC176018 | Hokkaido, Japan | Oya & Kajihara, (2017, 2020) |
|  | *Notocomplana koreana* (Kato, 1937) | LC508151 | LC508128 | LC176048 | LC176014 | Hokkaido, Japan | Oya & Kajihara, (2017, 2020) |
|  | *Notocomplana septentrionalis* (Kato, 1937) | LC508153 | LC508130 | LC176059 | LC176028 | Hokkaido, Japan | Oya & Kajihara, (2017, 2020) |
|  | *Notocomplana lapunda* (Marcus & Marcus,1968) | NA | MH700328 | NA | NA | Fort Pierce, Florida, USA | Litvaitis *et al*. (2019) |
| Notoplanidae | *Amyris hummelincki* Marcus & Marcus,1968 | NA | MH700269 | NA | NA | South Water Caye, Belize | Litvaitis *et al*. (2019) |
|  | *Notoplana australis* (Schmarda, 1859) | MW376750 | MW377503 | MW376595 | MW375907 | New South Wales, Australia | Rodríguez *et al*. (2021) |
|  | *Notoplana atomata* (Müller, 1776) | NA | MH700329 | NA | NA | Odiorne State Park, Rye, New Hampshire, USA | Litvaitis *et al*. (2019) |
|  | *Notoplana delicate* Yeri & Kaburaki, 1918 | LC508169 | LC508145 | LC508188 | LC508205 | Wakayama, Japan | Oya & Kajihara (2020) |
|  | *Notoplana queruca* Marcus & Marcus, 1968 | NA | MH700333 | NA | NA | Missouri Key, Florida, USA | Litvaitis *et al*. (2019) |
|  | *Notoplana felis* Rodríguez et al., 2021 | MW376753 | MW377506 | MW376598 | MW375910 | Victoria, Australia | Rodríguez *et al*. (2021) |
| Pseudostylochidae | *Pseudostylochus intermedius* Kato, 1939 | LC508164 | LC508141 | LC508183 | LC508201 | Aomori, Japan | Oya & Kajihara (2020) |
|  | *Pseudostylochus obscurus* (Stimpson, 1857) | LC508160 | LC508137 | LC508180 | LC508197 | Kanagawa, Japan | Oya & Kajihara (2020) |
|  | *Pseudostylochus elongatus* Kato, 1937 | LC508171 | LC508147 | NA | LC508207 | Kanagawa, Japan | Oya & Kajihara (2020) |
|  | *Pseudostylochus takeshitai* Yeri & Kaburaki, 1918 | LC508165 | LC508142 | LC508184 | LC508202 | Aomori, Japan | Oya & Kajihara (2020) |
|  | *Tripylocelis typica* Haswell, 1907 | MW376752 | MW377505 | MW376597 | MW375909 | New South Wales, Australia | Rodríguez *et al*. (2021) |
| Stylochoplanidae | *Armatoplana divae* (Marcus, 1947) | NA | MH700273 | NA | NA | Puerto Seco Beach Park, Jamaica | Litvaitis *et al*. (2019) |
|  | *Armatoplana kaburakii* Oya et al., 2022 | LC672051 | LC672050 | LC672053 | LC582946 | Misaki, Kanagawa, Japan | Oya *et al*. (2022) |
|  | *Armatoplana albomaculata* Oya et al., 2022 | LC672049 | LC672048 | LC672052 | LC672054 | Misaki, Kanagawa, Japan | Oya *et al*. (2022) |
|  | *Alloioplana yerii* Oya et al*.*, 2021 | LC651420 | LC651421 | NA | LC582944 | Misaki, Kanagawa, Japan | Oya *et al*. (2021) |
|  | *Comoplana agilis* (Lang, 1884) | MN334199 | MN384685 | NA | NA | Galicia, Spain | Dittmann *et al*. (2019) |
|  | *Comoplana pusilla* (Bock, 1924) | LC508157 | LC508134 | LC508177 | LC508194 | Hokkaido, Japan | Oya & Kajihara (2020) |
|  | *Phaenoplana kopepe* Oya & Kajihara, 2019 | LC508156 | LC508133 | LC508176 | LC369778 | Ogasawara Islands, Japan | Oya *et al.* 2019; Oya and Kajihara (2020) |
|  | *Stylochoplana clara* Kato, 1937 | MW376741 | MW377494 | MW376586 | MW375898 | Victoria, Australia | Rodríguez *et al*. (2021) |
| **Stylochoidea** |  |  |  |  |  |  |  |
|  | ***Cryptophallus sinensis* sp. nov.** | **PZ112129** | **PZ112134** | **PZ111958** | **PZ111956** | **Huidong, China** | **This study** |
| Callioplanidae | *Callioplana marginata* Stimpson, 1857 (1) | LC508159 | LC508136 | LC508179 | LC508196 | Kanagawa, Japan | Oya & Kajihara (2020) |
|  | *Neostylochus ancorus* Rodríguez et al., 2021 | MW376748 | MW377501 | MW376593 | MW375905 | New South Wales, Australia | Rodríguez *et al*. (2021) |
| Hoploplanidae | *Hoploplana californica* Hyman, 1953 | KC869797 | KC869850 | KC869753 | NA | California, USA | Laumer & Giribet, 2014 |
|  | *Hoploplana ornate* Yeri & Kaburaki, 1918 | LC508158 | LC508135 | LC508178 | LC508195 | Kanagawa, Japan | Oya & Kajihara (2020) |
| Latocestidae | *Latocestus* sp. | NA | MH700317 | NA | NA | South Water Caye, Belize | Litvaitis *et al*. (2019) |
|  | *Latocestus plehni* Laidlaw, 1906 | MZ292806 | MK299376 | MZ292852 | NA | Cape Verde Island | Cuadrado *et al*. (2024) |
|  | *Eulatocestus australis* Rodríguez et al., 2021 | MW376749 | MW377502 | MW376594 | MW375906 | New South Wales, Australia | Rodríguez *et al*. (2021) |
| Limnostylochidae | ***Limnoplana obscuriviridis* sp. nov.** | **PZ112131** | **PZ112136** | **PZ111960** | **PZ111957** | **Shenzhen, China** | **This study** |
| Planoceridae | *Heteroplanocera katoi* Oya & Kajihara, 2021 | LC545566 | LC545568 | LC545564 | LC545562 | Shizuoka, Japan | Oya & Kajihara (2021) |
|  | *Paraplanocera marginata* Meyer, 1922 | MW376745 | MW377498 | MW376590 | MW375902 | New South Wales, Australia | Rodríguez *et al*. (2021) |
|  | *Paraplanocera oligoglena* (Schmarda,1859) | LC545567 | LC545569 | LC545565 | LC545563 | Kagoshima, Japan | Oya & Kajihara (2021) |
|  | *Planocera multitentaculata* Kato, 1944 | LC508150 | LC508127 | LC508174 | LC508192 | Kanagawa, Japan | Oya & Kajihara (2020) |
|  | *Planocera pellucida* (Mertens, 1833) | MN334203 | MN384696 | NA | NA | Canary Islands, Spain | Dittmann *et al*. (2019) |
|  | *Planocera reticulata* (Stimpson, 1855) | LC508172 | LC508148 | LC508190 | LC508208 | Kagoshima, Japan | Oya & Kajihara (2020) |
|  | *Planocera edmondsi* Prudhoe, 1982 | MW376755 | MW377508 | MW376600 | MW375912 | Victoria, Australia | Rodríguez *et al*. (2021) |
| Plehniidae | *Paraplehnia pacifica* (Kato, 1939) | LC508155 | LC508132 | LC508175 | LC508193 | Aomori, Japan | Oya & Kajihara (2020) |
|  | *Paraplehnia seisuiae* Oya et al., 2019 | LC508167 | LC467000 | LC508186 | LC466999 | Mie, Japan | Oya & Kajihara (2020) |
| Idioplanidae | *Idioplana atlantica* (Bock, 1913) | NA | MH700310 | NA | NA | Crawl (Kraal) Cay, Bocas del Toro, | Litvaitis *et al*. (2019) |
|  | *Idioplana australiensis* Woodworth, 1898 | MW376746 | MW377499 | MW376591 | MW375903 | New South Wales, Australia | Rodríguez *et al*. (2021) |
| Stylochidae | *Stylochus* cf. *Aomori* (Kato, 1937) | LC508163 | LC508140 | LC508182 | LC508200 | Aomori, Japan | Oya & Kajihara (2020) |
|  | *Stylochus fafai* (Marquina et al., 2015) | MZ292817 | MZ292835 | MZ292865 |  | Asturias, Spain | Cuadrado *et al*. (2024) |
|  | *Stylochus neapolitanus* (Delle Chiaje, 1841) | MZ292800 | MZ292841 | MZ292846 |  | Galicia, Spain | Cuadrado *et al*. (2024) |
|  | *Stylochus zebra* (Verrill 1882) | AF342801 | AF342800 | NA | NA | US Atlantic coast | Dittmann *et al*. (2019) |
|  | *Stylochus stellae* (Marquina et al., 2014) | MN334201 | MN384692 | NA | NA | Valencia, Spain | Dittmann *et al*. (2019) |
|  | *Leptostylochus* cf. *gracilis* Kato, 1934 | LC508161 | LC508138 | LC508181 | LC508198 | Kanagawa, Japan | Oya & Kajihara (2020) |
|  | *Leptostylochus victoriensis* Beveridge, 2017 | MW376742 | MW377495 | MW376587 | MW375899 | New South Wales, Australia | Rodríguez *et al*. (2021) |
|  | *Mirostylochus akkeshiensis* Kato, 1937 | LC508173 | LC508149 | LC508191 | LC508209 | Hokkaido, Japan | Oya & Kajihara (2020) |
| Outgroup |  |  |  |  |  |  |  |
|  | *Cestoplana rubrocincta* (Grube, 1840) | MW376751 | MW377504 | MW376596 | MW375908 | New South Wales, Australia | Rodríguez *et al*. (2021) |
|  | *Pericelis tectivorum* Dittmann et al., 2019 | MN334202 | MK181524 |  |  | Aquaria Innsbruck, Austria | Dittmann *et al*. (2019) |

**References**

Bahia J, Padula V, Schrödl M (2017) Polycladida phylogeny and evolution: integrating evidence from 28S rDNA and morphology. Organisms, Diversity and Evolution 17: 653–678. https://doi.org/10.1007/s13127-017-0327-5

Cuadrado D, Rodríguez J, Machordom A, Noreña C, Fernández-Álvarez FÁ, Hutchings P, Williamson J (2024) Base-substitution rates of nuclear and mitochondrial genes for polyclad flatworms. Zoosystematics and Evolution 100: 863–876. https://doi.org/10.3897/zse.100.119945

Dittmann IL, Cuadrado D, Aguado MT, Noreña C, Egger B (2019) Polyclad phylogeny persists to be problematic. Organisms, Diversity and Evolution: 1–24. https://doi.org/10.1007/s13127-019-00415-1

Litvaitis MK, Bolaños DM, Quiroga SY (2019) Systematic congruence in Polycladida (Platyhelminthes, Rhabditophora): are DNA and morphology telling the same story? Zoological Journal of the Linnean Society 20: 1–27. https://doi.org/10.1093/zoolinnean/zlz007

Oya Y, Kajihara H (2017) Description of a new *Notocomplana* species (Platyhelminthes: Acotylea), new combination and new records of Polycladida from the northeastern Sea of Japan, with a comparison of two different barcoding markers. Zootaxa 4282(3): 526–542. https://doi.org/10.11646/zootaxa.4282.3.6

Oya Y, Kajihara H (2020) Molecular phylogenetic analysis of Acotylea (Platyhelminthes: Polycladida). Zoological Science 37(3): 271–279. https://doi.org/10.2108/zs190136

Oya Y, Kajihara H (2021) Description and phylogenetic relationships of a new genus of Planoceridae (Polycladida, Acotylea) from Shimoda, Japan. Journal of the Marine Biological Association of the United Kingdom 101(1): 81–88. https://doi.org/10.1017/s0025315421000060

Oya Y, Kimura T, Kajihara H (2019) Description of a new species of Paraplehnia (Polycladida, Stylochoidea) from Japan, with inference on the phylogenetic position of Plehniidae. ZooKeys 864: 1–13. https://doi.org/10.3897/zookeys.864.33955

Oya Y, Tsuyuki A, Kajihara H (2020) Descriptions of two new species of *Armatoplana* (Polycladida: Stylochoplanidae) from the coasts of Japan, with their phylogenetic positions in Leptoplanoidea. Zootaxa 5178 (5): 433–452. https://doi.org/10.11646/zootaxa.5178.5.2

Rodríguez J, Hutchings PA, Williamson JE (2021) Biodiversity of intertidal marine flatworms (Polycladida, Platyhelminthes) in southeastern Australia. Zootaxa 5024: 1–63. https://doi.org/10.11646/zootaxa.5024.1.1


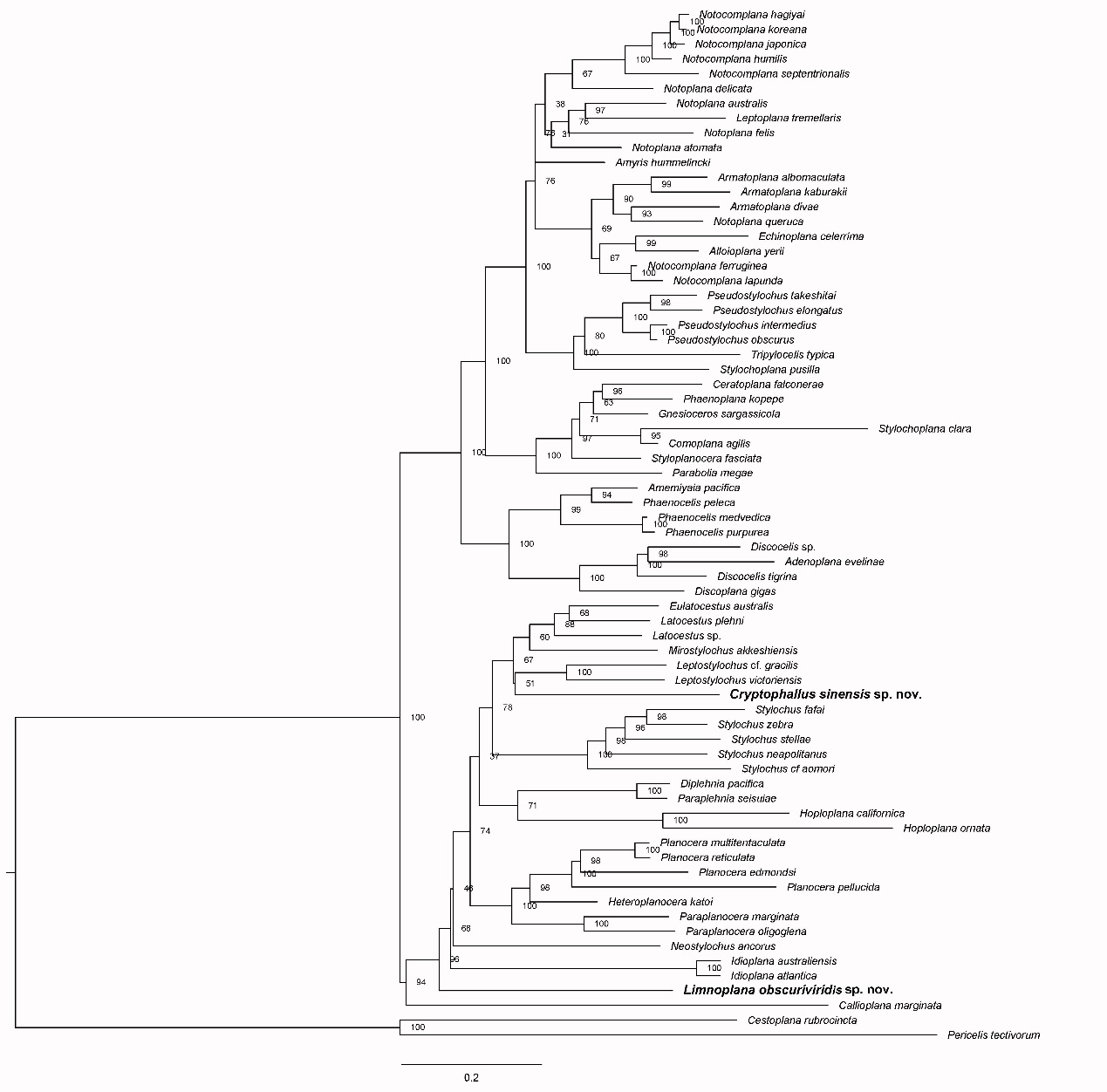


**Figure S1.** Maximum-likelihood tree based on a concatenated dataset of partial 18S, 28S, 16S and COI sequences.
